# Evaluation of the clinical efficacy of Ru’ai Shuhou recipe for the prevention of lung metastases from breast cancer: a retrospective study based on propensity score matching

**DOI:** 10.3389/fphar.2024.1406862

**Published:** 2024-08-02

**Authors:** Nan-Nan Du, Shi-Jun Shao, Jia-Mei Feng, Hua Wan, Xue-Qing Wu

**Affiliations:** Breast Department, Shuguang Hospital Affiliated to Shanghai University of Traditional Chinese Medicine, Shanghai, China

**Keywords:** Ru’ai Shuhou recipe, breast cancer, long-term prognosis, cohort study, survival analysis, propensity score matching

## Abstract

**Background:**

Breast cancer lung metastasis occurs at a high rate and at an early stage, and is the leading cause of death in breast cancer patients. The aim of this study was to investigate the effect of Ru’ai Shuhou Recipe (RSR) intervention on the occurrence of recurrent metastases, especially lung metastases, in postoperative patients with breast cancer.

**Materials and Methods:**

A retrospective cohort study was implemented at Shuguang Hospital of Shanghai University of Traditional Chinese Medicine in China between January 2014 to January 2019. Female patients were included according to the propensity score matching (PSM) method and balanced on the basis of general and clinical information such as age, body mass index, neo-adjuvant therapy, and surgical approach. Patients with pathological diagnosis of breast cancer were included in this study. Breast cancer patients were divided into exposed and non-exposed groups according to whether they took RSR-based botanical drugs after surgery. Kaplan-Meier survival analysis and Cox survival analysis to explore the relationship between RSR and 5-year disease-free survival and incidence of lung metastases in breast cancer patients after surgery.

**Results:**

360 female patients were assessed and 190 patients were included in the study after PSM (95 in each of the exposed and non-exposed groups). Of the 190 patients after PSM, 55.79% were over 50 years of age. The mean follow-up time was 60.55 ± 14.82 months in the exposed group and 57.12 ± 16.37 months in the non-exposed group. There was no significant baseline characteristics difference between two groups. Kaplan-Meier analysis showed that the 5-year incidence of lung metastases was significantly lower in the exposed group, and the disease-free survival of patients was significantly longer. Cox univariate and multivariate analysis showed that neoadjuvant chemotherapy and lymph node metastasis were independent risk factors for the development of breast cancer lung metastasis, with risk ratios of 17.188 and 5.812, while RSR treatment was an independent protective factor against the development of breast cancer lung metastasis, with a risk ratio of 0.290.

**Conclusion:**

Standard biomedical treatment combined with RSR intervention can better prevent breast cancer recurrence and metastasis, reduce the incidence of lung metastasis in patients, and improve long-term prognosis.

## Introduction

Breast cancer is currently the most prevalent cancer in women, and has continued to increase at an average rate of 0.5% per year over the past 10 years (Giaquinto et al., 2022). In 2020, there are approximately 2.3 million new cases of breast cancer and 685,000 deaths worldwide. China accounts for 18% of new cases and 17% of cancer-related deaths worldwide (Siegel et al., 2023). Recurrent metastases, which can occur years to decades after diagnosis of the primary tumour, are the leading cause of death in breast cancer patients, accounting for approximately 90% of breast cancer-related deaths (Medeiros and Allan, 2019).

Breast cancer can metastasise to the bones, brain, liver and lungs, depending on the organ’s predisposition to metastasis. A study based on the SEER database, which included 17,455 cases of recurrent metastases, showed that among breast cancer patients with metastases, 30%–60% had lesions in the bone, 4%–10% in the brain, 15%–32% in the liver, 21%–32% in the lungs (Wu et al., 2017). Lung metastases are of particular concern because of the early onset of the disease and the high risk of death. Studies have shown that lung metastases usually occur within 5 years of breast cancer diagnosis and are strongly associated with death in 60%–70% of breast cancer patients (Medeiros and Allan, 2019). In the metastatic cascade, the lung is the first major capillary bed encountered by breast cancer cells after they escape into the bloodstream, with a vascular surface area of up to 100 m^2^, providing a great opportunity for retention and extravasation of breast cancer cells with diameters five times greater than those of the pulmonary capillary bed (MacDonald et al., 2002; Stott et al., 2010). As a result, the lung is usually the first and only site of metastasis in about 25% of breast cancer patients (Berman et al., 2013). On the other hand, despite the current boom in medical development, local surgery, chemotherapy, radiotherapy, radiofrequency ablation and endocrine therapy can improve the prognosis of metastatic breast cancer, prolonging patients’ disease-free survival and overall survival, the prognosis for patients with lung metastases from breast cancer is still poor, with a median survival of only 25 months (EL Baiomy and El Kashef, 2017; Macherey et al., 2017; Xiao et al., 2018; Li J. et al., 2019; Waks and Winer, 2019; Kazmi et al., 2020; Kroeze et al., 2023). Lung metastases continue to disrupt normal lung function, leading to coughing, haemoptysis, breathlessness and eventually death. Therefore, finding therapeutic strategies to improve the prognosis of breast cancer lung metastases is an important measure to improve the overall survival of patients with breast cancer metastases, and it is also an important problem that needs to be solved urgently.

The current research is primarily focused on understanding the molecular mechanisms that drive breast cancer metastasis to the lung and, based on this, identifying potential/emerging therapeutic approaches. The primary tumour can “trigger” or enhance the microenvironment of distant organs in preparation for metastasis, a process known as the pre-metastatic niche (PMN). Lung colonisation is facilitated by a complex web of interactions with the tumour microenvironment, lung stroma, immune cells and MDSCs, and crosstalk between these components is mediated by exosomes and tumour/stroma-derived factors (Medeiros and Allan, 2019). Targeting PMN is an effective means of preventing the development of lung metastases (Li Z. et al., 2019). Lee et al. found that LSD1-specific inhibitors remodeled bone marrow compartments in a model of spontaneous lung metastasis, and that the mechanism was related to inhibition of MDSCs infiltration of the primary tumour and lung by CCL2, which ultimately shrinks metastatic lesions in the lung (Lee et al., 2018). Bone marrow cells can be recruited to the lungs by CSF-1-containing exosomes produced under hypoxic conditions, a process inhibited by GW2580 (Borin et al., 2017). Pretreatment of mice with GW2580 prior to tumour implantation significantly reduced bone marrow cell recruitment to the lungs and increased the number of anti-tumour M1 macrophages. However, these studies remain in the pre-clinical setting and there are no targeted strategies for the early detection or eradication of breast cancer lung metastases.

**FIGURE 1:**
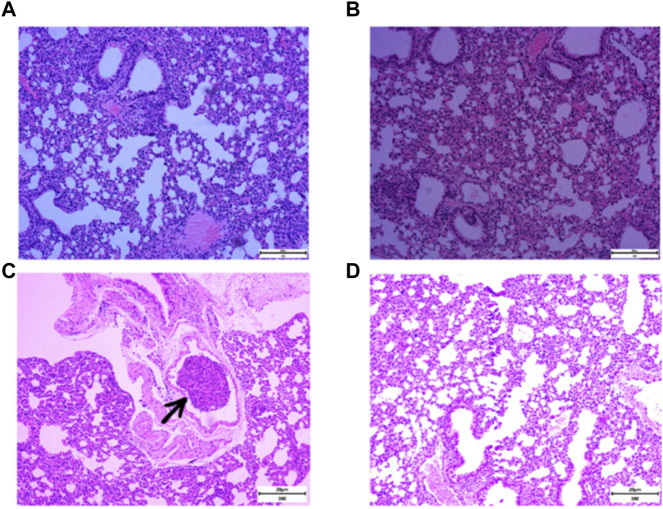
Incidence of lung metastases in 4T1 breast cancer mice after normal saline (NS) and RSR treatment by gavage. [**(A and B)**, no lung metastases were seen in mice given NS and RSR by gavage for 1 week; **(C)**, lung metastases were seen in mice given NS by gavage for 3 weeks; and **(D)**, no lung metastases were seen in mice given RSR by gavage for 3 weeks)].

RSR was created by Professor Lu Deming, a famous practitioner of Chinese medicine in Shanghai, for the characteristics of post-operative recurrence and metastasis of breast cancer. In the formula, Yinyanghuo and Shanzhuyu nourish the kidneys and replenish the innate, Huangqi, Baizhu and Fuling nourish the qi and strengthen the spleen to replenish the nourishment, while Ezhu and Fengfang invigorate the blood and detoxify the toxin to drive away evil spirits. This formula has been used clinically for more than 30 years and is effective in prolonging the disease-free survival and overall survival of patients (Qu et al., 2012; Gao et al., 2022; Wu et al., 2000). Animal experiments have found that RSR can significantly reduce the incidence of lung metastasis in mice with 4T1 lung high metastatic breast cancer, and the earlier the herbal intervention, the lower the chance of distant metastasis occurred (Wu et al., 2010; Ding et al., 2022). Typically, 4T1 breast cancer cells can be detected in the lungs 14 days after inoculation and can form pathologically visible metastases in the lungs by 21 days (Yan et al., 2010). As shown in Figure 1, RSR significantly inhibited the development of lung metastases in breast cancer mice after 3 weeks. Unfortunately, the inhibitory effect of RSR on breast cancer lung metastasis has not yet been evaluated under real-world conditions, nor have the benefits of integrated modulation of interventional efficacy, pleiotropic effects and improvement of long-term prognosis been demonstrated. Therefore, 360 breast cancer patients operated at Shuguang Hospital of Shanghai University of Traditional Chinese Medicine were selected for this study to conduct a cohort study for long-term follow-up. Using propensity score matching (PSM) for accurate comparison, the relationship between RSR intervention and lung metastasis was explored to provide clinical evidence for traditional chinese medicine in the prevention and treatment of breast cancer lung metastasis.

## Methods

### Patients

A retrospective cohort study was performed at Shuguang Hospital of Shanghai University of Traditional Chinese Medicine in China between January 2014 to January 2019. Female patients were included through strict inclusion and exclusion criteria screening.

The current study conformed to the principles drafted in the Helsinki declaration and was approved by the medical ethical committee of Shuguang Hospital Affiliated to Shanghai University of Traditional Chinese Medicine (approval number: 2024-1505-088-01). Informed consent was obtained from all patients.

**Inclusion criteria:** 1) Patients with spleen and kidney deficiency in breast cancer who meet the diagnostic criteria; 2) Age at the time of surgery 18–75 years; 3) All of them received standardised treatment (surgery ± chemotherapy ± radiotherapy ± endocrine therapy ± targeted therapy); 4) If herbal treatment is used, it must be started within 1 year of surgery; 5) All patients must be treated for ≥18 months, unless metastases recur during herbal treatment.

**Exclusion criteria:** 1) Recurrence or metastasis before RSR was taken in the exposure group; 2) Recurrence or metastasis during radiotherapy or chemotherapy in the non-exposed group; 3) Those who have incomplete clinical information.

**Grouping and treatment:** The non-exposed group was treated with standard biomedical treatment according to the National Comprehensive Cancer Network (NCCN) guidelines in the year of surgery (Table 1); the exposed group was treated with RSR in addition to the treatment in the non-exposed group (Table 2).

**TABLE 1:** Post-operative adjuvant therapy regimens.

Items	Numbers (%)	Dosing
Chemotherapy		
AC	44 (13.17%)	Doxorubicin 60 mg/m^2^ IV on day 1
		Cyclophosphamide 600 mg/m^2^ IV day 1
		Cycled every 21 days for 4 cycles
AC-T	65 (19.46%)	Doxorubicin 60 mg/m^2^ IV on day 1
		Cyclophosphamide 600 mg/m^2^ IV day 1
		Cycled every 21 days for 4 cycles
		Followed by
		Docetaxel 100 mg/m^2^ IV on day 1
		Cycled every 21 days for 4 cycles
TC	81 (24.25%)	Docetaxel 75 mg/m^2^ IV day 1
		Cyclophosphamide 600 mg/m^2^ IV day 1
		Cycled every 21 days for 4 cycles
EC-T	68 (20.36%)	Epirubicin 100 mg/m^2^ IV day 1
		Cyclophosphamide 830 mg/m^2^ IV day 1
		Cycled every 21 days for 4 cycles
		Followed by
		Docetaxel 100 mg/m^2^ IV on day 1
		Cycled every 21 days for 4 cycles
Others	76 (22.75%)	—
Targeted therapy		
Trastuzumab	64 (94.12%)	Trastuzumab 8 mg/kg IV day 1
		Followed by
		Trastuzumab 6 mg/kg IV on day 1
		Cycled every 21 days to complete 1 year of therapy
		Trastuzumab 8 mg/kg IV day 1
		Pertuzumab 840 mg IV day 1
Trastuzumab + Pertuzumab	4 (5.88%)	Followed by
		Trastuzumab 6 mg/kg IV on day 1
		Pertuzumab 420 mg IV day 1
		Cycled every 21 days to complete 1 year of therapy
Endocrine therapy		
Anastrozole	32 (12.45%)	1 mg/d orally for 5 years
Toremifene	58 (22.57%)	60 mg/d orally for 5 years
Letrozole	89 (34.63%)	2.5 mg/d orally for 5 years
Tamoxifen	45 (17.51%)	20 mg/d orally for 5 years
Exemestane Tablets	33 (12.84%)	25 mg/d orally for 5 years
**Radiotherapy**	189 (52.50%)	Whole Breast Radiation: 40–42.5 Gy in 15–16 fractions; in selected cases 45–50.4 Gy in 25–28 fractions may be considered
		Chest Wall Radiation: 45–50.4 Gy at 1.8–2 Gy/fx
		Regional Nodal Radiation: 45–50.4 Gy at 1.8–2 Gy/fx

Abbreviations: A, doxorubicin; C, cyclophosphamide; T, docetaxel; E, epirubicin; IV, intravenous injection.

**TABLE 2:** Traditional herbal remedies of RSR and their modification according to symptoms.

	Family	TCM herbs	Original plants	Part(s) of plant used
The formula of RSR	Fabaceae	Huangqi	Astragalus membranaceus (Fisch.) Bge.var.mongholicus (Bge.) Hsiao and Astragalus membranaceus (Fisch.) Bge	Dried rhizomes
	Asteraceae	Baizhu	Atractylodes macrocephala Koidz	Dried rhizomes
	Polyporaceae	Fuling	Poria cocos (Schw.) Wolf	Dried sclerotia
	Berberidaceae	Yinyanghuo	Epimedium brevicornu Maxim、Epimedium sagittatum (Sieb. et Zucc.) Maxim.、Epimedium pubescens Maxim. and Epimedium koreanum Nakai	Dried aerial parts
	Cornaceae	Shanzhuyu	Cornus officinalis Sieb. et Zucc	Dried ripe sarcocarp
	Zingiberaceae	Ezhu	Curcuma phaeocaulis VaL.、Curcuma kwangsiensis S.G.Lee et C.F.Liang and Curcuma wenyujin Y.H.ChenetC.Ling	Dried root tuber
	Vespidae	Fengfang	Polistes olivaceous (DeGeer) Polistes japonicus Saussure and Parapolybia varia Fabricius	Nest
Accompanied by lumbar and knee pain and aching limbs	Fabaceae	Buguzhi	Psoralea corylifolia L	Dried ripe fruit
	Eucommiaceae	Duzhong	Eucommia ulmoides Oliv	Dried stem bark
	Amaranthaceae	Niuxi	Achyranthes bidentata Bl	Dried root
	Santalaceae	Hujisheng	Viscum coloratum (Komar.) Nakai	Dried stem and branch with leaf
Accompanied by easy awakening at night and insomnia	Rhamnaceae	Suanzaoren	Ziziphus jujuba Mill. var. spinosa (Bunge) Hu ex H. F. Chou	Dried ripe fruit
	Polygalaceae	Yuanzhi	Polygala tenuifolia Willd. and Polygala sibirica L	Dried root
	Polyporaceae	Lingzhi	Ganoderma lucidum (Leyss.ex Fr.) Karst. and Ganoderma sinense Zhao, Xu et Zhang	Dried fruiting body
	Polygonaceae	Shouwuteng	Polygonum multiflorum Thunb	Dried lianoid stem
With nausea and vomiting, loss of appetite	Poaceae	Zhuru	Bambusa tuldoides Munro、Sinocalamus beecheyanus (Munro)McClure var. Pubescens P.F.Li、Phyllostachys nigra (Lodd.) Munro var.henonis (Mitf.) Stapf ex Rendle	Dried middle shavings of stem
	Araceae	Jiangbanxia	Pinellia ternata (Thunb.) Breit	Dried tuber
	Zingiberaceae	Shengjiang	Zingiber officinale Rosc	Freshed rhizome
	Ranunculaceae	Huanglian	Coptis chinensis Franch.、Coptis deltoidea C.Y.Cheng et Hsiao and Coptis teeta Wall	Dried rhizome
	Rutaceae	Wuzhuyu	Euodia rutaecarpa (Juss.) Benth.、Euodia rutaecarpa (Juss.) Benth. var. officinalis (Dode) Huang and Euodia rutaecarpa (Juss.) Benth. var. bodinieri (Dode) Huang	Dried and nearly ripe fruit
Sweating with heat, night sweats	Poaceae	FuXiaomai	*Triticum aestivum* L	Dried blighted caryopsis
	Fabaceae	Gancao	Glycyrrhiza uralensis Fisch.、Glycyrrhiza inflata Bat. and Glycyrrhiza glabra L	Dried root and rhizome
	Rhamnaceae	Dazao	Ziziphus jujuba Mill	Dried ripe fruit
	Asparagaceae	Zhimu	Anemarrhena asphodeloides Bge	Dried rhizome
	Rutaceae	Huangbo	Phellodendron chinense Schneid	Dried bark
With palpitations	Asparagaceae	Maidong	Ophiopogon japonicus (L.f) Ker-Gawl	Dried root tuber
	magnoliaceae	Wuweizi	Schisandra chinensis (Turcz.) Baill	Dried ripe fruit
	Fabaceae	Zhigancao	Glycyrrhiza uralensis Fisch.、Glycyrrhiza inflata Bat. and Glycyrrhiza glabra L	Dried root and rhizome
	Lauraceae	Guizhi	Cinnamomum cassia Presl	Dried young branches
Accompanied by constipation	Moraceae	Huomaren	Cannabis sativa L	Dried ripe fruit
	Scrophulariaceae	Dihuang	Rehmannia glutinosa Libosch	Dried root tuber
	Asteraceae	Shengbaizhu	Atractylodes macrocephala Koidz	Dried rhizome
	Asphodelaceae	Luhui	Aloe barbadensis Miller、Aloe ferox Miller	Dried latex
Improving anti-tumour efficacy	Selaginellaceae	Juanbai	Selaginella tamariscina (Beauv.) Spring and Selaginella pulvinata (Hook, etGrev.) Maxim	Dried herb
	Solanaceae	Longkui	Solanum nigrum L	Dried whole plant
	Lamiaceae	Banzhilian	Scutellaria barbata D.Don	dried herb

RSR, was provided by the Chinese herbal pharmacy of Shuguang Hospital, one dose per day, routinely decocted into 300 mL and taken orally twice in the morning and evening, 150 mL each time. The formula of RSR, is (addition and subtraction of homemade formula): Huangqi 10 g (g), Baizhu 6g, Fuling 6g, Yinyanghuo 10g, Shanzhuyu 6g, Ezhu 10g, Fengfang 10 g.

Accompanied by lumbar and knee pain and aching limbs: Add Buguzhi 12g, Duzhong 12g, Niuxi 12 g and Hujisheng 12g; Accompanied by easy awakening at night and insomnia: Add Suanzaoren 12g, Yuanzhi 9g, Lingzhi 15g, Shouwuteng 15g; With nausea and vomiting, loss of appetite: Add Zhuru 9g, Jiangbanxia 9g, Shengjiang 3g, Huanglian 6g, Wuzhuyu 3g; Sweating with heat, night sweats: Add Xiaomai 30g, Gancao 9g, Dazao 12g, Zhimu 12g, Huangbo 9g; With palpitations: Add Maidong 12g, Wuweizi 12g, Zhigancao 9g, Guizhi 12g; Accompanied by constipation: Add Huomaren 30g, Dihuang 15g, Baizhu 30g, Luhui 15g; Improving anti-tumour efficacy: Add Juanbai 15g, Longkui 30g, Banzhilian 30g (Table 2).

Serum liver and kidney function tests were performed every 3 months during the oral administration of traditional Chinese medicine in the exposed group to exclude adverse drug reactions.

### Evaluation and follow-up

Patients were evaluated for recurrent metastases: 6 months/time within 2 years after surgery and 12 months/time after 2 years.

### Outcomes

The primary outcome was lung metastases-free survival. The criteria for determining lung metastases were based on imaging studies, such as CT or MRI, combined with medical history and clinical diagnostic criteria. A 5-year incidence of lung metastasis = number of cases of lung metastasis in 5 years/total number of cases included. The secondary outcomes were disease-free survival (DFS). Disease-free survival is defined as the time from surgical treatment to tumour progression, recurrence, metastasis or death (The Society of Breast Cancer China Anti-Cancer Association, 2015). 5-year disease-free survival = number of cases without recurrent metastases/total number of cases included.

### Statistical analysis

Propensity score matching (PSM) is a statistical matching technique that attempts to reduce the bias caused by differences in covariates in the study. In the analysis of observational data, bias could arise because of lack of randomization. PSM creates a sample of units in different groups that are comparable on all observed covariates to mimic randomization and reduce potential bias. In our study, PSM was performed between patients who received and not received RSR therapy. Matching was done based upon age, body mass index (BMI), neo-adjuvant chemotherapy (NAC, Yes vs. No), breast conservation (Yes vs. No), invasive ductal carcinoma (IDC, Yes vs. No), histopathological grade (1 vs. 2 vs. 3), estrogen receptor status (ER, negative vs. 1%–10% vs. >10%), progesterone receptor status (PR, negative vs. 1%–10% vs. 10%), human epidermal growth factor receptor-2 status (HER2, negative vs. positive), Ki67 status (≤ 15% vs. 15%–30% vs. >30%), axillary lymph node metastasis (ALN, yes vs. no), chemotherapy (yes vs. no), targeted therapy (yes vs. no), endocrine therapy (yes vs. no) and radiotherapy (yes vs. no) using a 1:1 nearest-neighbor method without replacement. The caliper width was equal to 0.2 times the logit standard deviation of the propensity score. After matching, the statistical significance and standardized differences in the covariate balance were reviewed. Univariable analyses of lung metastases-free survival and disease-free survival were conducted using the Kaplan-Meier method, and the log-rank test was used for group comparisons. Graph processing software GraphPad Prism was applied for statistical analysis and graphing. Cox survival analysis was performed to explore the correlation between breast cancer lung metastases and age, BMI, NAC, breast conservation, histopathological grade, ER, PR, HER2, Ki67, ALN, chemotherapy, targeted therapy, endocrine therapy, radiotherapy and RSR intervention. Data collection and statistical analysis were performed using IBM SPSS Statistic 24.0 (IBM Corporation, Armonk, NY), with *p*-values, and 95% confidence intervals (*CI*) calculated for each model. All tests were two-sided, and significance was set at *p* < 0.05.

## Results

### Baseline characteristics between two groups were well balanced following PSM

**FIGURE 2:**
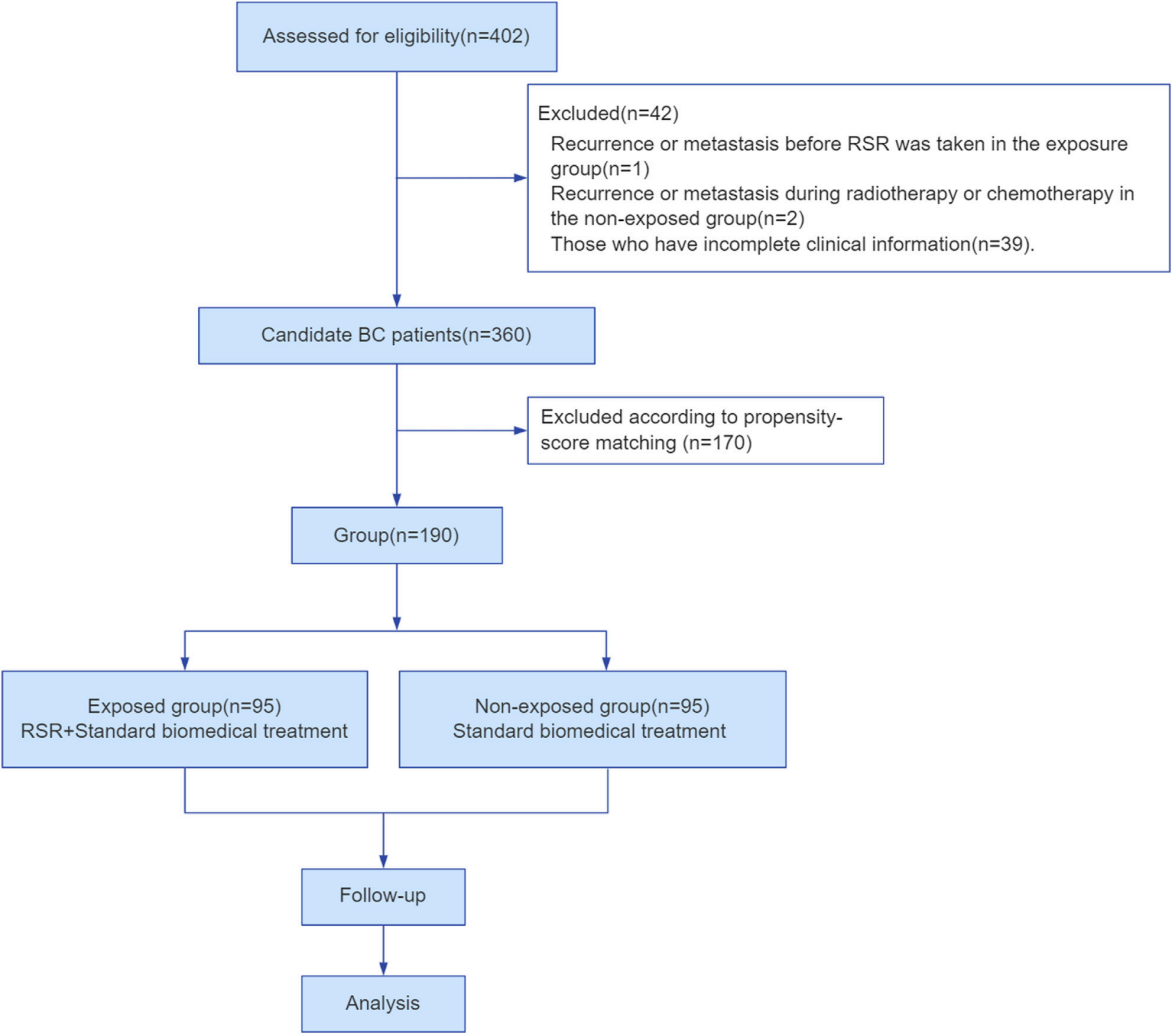
Flow diagram of the trial.

In total, 402 female breast cancer patients were employed at Shuguang Hospital of Shanghai University of Traditional Chinese Medicine in China between January 2014 and January 2019. Among them, 360 patients met the inclusion and exclusion criteria. Finally, 190 cases were included and divided into two groups according to the matching propensity score in this study (Figure 2).

The clinical data between the two groups before and after matching the propensity score were analyzed, including age, body mass index (BMI), neo-adjuvant chemotherapy (NAC), breast conservation, invasive ductal carcinoma (IDC), histopathological grade, estrogen receptor (ER) status, progesterone receptor (PR) status, human epidermal growth factor receptor-2 (HER2) status, axillary lymph node (ALN) metastasis, chemotherapy, targeted therapy, endocrine therapy and radiotherapy (Table 3). Before the tendency matching, the proportion of patients over 50 years of age in the exposed group was higher compared to the non-exposed group (52.04% vs 66.46%, *p* = 0.008). Additionally, the exposed group had a lower proportion of patients who underwent breast-conserving surgery, chemotherapy, radiotherapy and those with positive HER2 expression (53.57% vs 25.61%, *p* = 0.001; 98.47% vs 85.98%, *p* = 0.001; 64.80% vs 31.63%, *p* = 0.001; 26.53% vs 17.07%, *p* = 0.043) (Table 3). After the PSM, the primary conditions of patients between the two groups were balanced, and the difference was not significant.

**TABLE 3:** Baseline characteristics and propensity score matching.

Variable	Prematching (n = 360)				Postmatching (n = 190)			
	Overall (n = 360)	Non-exposed group (n = 196)	Exposed group (n = 164)	P	Overall (n = 190)	Non-exposed group (n = 95)	Exposed group (n = 95)	P
Age (n,%)								
<50	149 (41.39)	94 (47.96)	55 (33.54)	0.008	84 (44.21)	43 (45.26)	41 (43.16)	0.770
≥50	211 (58.61)	102 (52.04)	109 (66.46)		106 (55.79)	52 (54.74)	54 (56.84)	
BMI (n,%)								
<25	240 (66.67)	129 (65.82)	111 (67.68)	0.793	142 (74.74)	72 (75.79)	70 (73.68)	0.738
≥25	120 (33.33)	67 (34.18)	53 (32.32)		48 (25.26)	23 (24.21)	25 (26.32)	
NAC (n,%)								
Yes	28 (7.78)	18 (9.18)	10 (6.10)	0.373	13 (6.84)	8 (8.42)	5 (5.26)	0.389
No	332 (92.22)	178 (90.82)	154 (93.90)		177 (93.16)	87 (91.58)	90 (94.74)	
Breast conservation (n,%)								
Yes	147 (40.83)	105 (53.57)	42 (25.61)	0.001	64 (37.85)	34 (33.68)	30 (35.79)	0.539
No	213 (59.17)	91 (46.43)	122 (74.39)		126 (62.15)	61 (66.32)	65 (62.62)	
IDC (n,%)								
Yes	347 (96.39)	191 (97.45)	156 (95.12)	0.371	184 (96.84)	91 (95.79)	93 (97.89)	0.407
No	13 (3.61)	5 (2.55)	8 (4.88)		6 (3.16)	4 (4.21)	2 (2.11)	
Chemotherapy (n,%)								
Yes	334 (92.78)	193 (98.47)	141 (85.98)	0.001	187 (98.42)	93 (97.89)	94 (98.95)	0.561
No	26 (7.22)	3 (1.53)	23 (14.02)		3 (1.58)	2 (2.11)	1 (1.05)	
Targeted therapy (n,%)								
Yes	68 (18.89)	44 (22.45)	24 (14.63)	0.140	37 (19.47)	17 (17.89)	20 (21.05)	0.775
No	292 (81.11)	152 (77.55)	140 (85.37)		153 (80.53)	78 (82.11)	75 (78.95)	
Endocrine therapy (n,%)								
Yes	255 (70.83)	134 (68.37)	121 (73.78)	0.260	130 (68.42)	67 (70.53)	63 (66.32)	0.532
No	105 (29.17)	62 (31.63)	43 (26.22)		60 (31.58)	28 (29.47)	32 (33.68)	
Radiotherapy (n,%)								
Yes	189 (52.5)	127 (64.80)	62 (31.63)	0.001	102 (53.68)	54 (56.84)	48 (50.53)	0.245
No	171 (47.5)	69 (35.20)	102 (52.04)		88 (46.32)	41 (43.16)	47 (49.47)	
Histopathological grade (n,%)								
1	8 (2.22)	6 (3.06)	2 (1.22)	0.488	5 (2.63)	4 (4.21)	1 (1.05)	0.396
2	198 (55.00)	105 (53.57)	93 (56.71)		98 (51.58)	48 (50.53)	50 (52.63)	
3	154 (42.78)	85 (43.37)	69 (42.07)		87 (45.79)	43 (45.26)	44 (46.32)	
ER (n,%)								
Negative	101 (28.06)	56 (28.57)	45 (27.44)	0.057	60 (31.58)	28 (29.47)	32 (33.68)	0.617
1%–10%	23 (6.39)	7 (3.57)	16 (9.76)		6 (3.16)	4 (4.21)	2 (2.11)	
>10%	236 (65.56)	133 (67.86)	103 (62.80)		124 (65.26)	63 (66.32)	61 (64.21)	
PR (n,%)								
Negative	166 (46.11)	94 (47.96)	72 (43.90)	0.710	93 (48.95)	47 (49.47)	46 (48.42)	0.924
1%–10%	59 (16.39)	32 (16.33)	27 (16.46)		30 (15.79)	14 (14.74)	16 (16.84)	
>10%	135 (37.50)	70 (35.71)	65 (39.63)		67 (35.26)	34 (35.79)	33 (34.74)	
HER2 (n,%)								
Negative	280 (77.78)	144 (73.47)	136 (82.93)	0.043	147 (77.37)	76 (80.00)	71 (74.74)	0.386
Positive	80 (22.22)	52 (26.53)	28 (17.07)		43 (22.63)	19 (20.00)	24 (25.26)	
KI67 (n,%)								
≤15%	88 (24.44)	52 (26.53)	36 (21.95)	0.588	42 (22.11)	20 (21.05)	22 (23.16)	0.409
15%–30%	106 (29.44)	57 (29.08)	49 (29.88)		48 (25.26)	28 (29.47)	20 (21.05)	
>30%	166 (46.11)	87 (44.39)	79 (48.17)		100 (52.63)	47 (49.47)	53 (55.79)	
ALN (n,%)								
Negative	213 (59.17)	113 (57.65)	100 (60.98)	0.595	104 (54.74)	54 (56.84)	50 (52.63)	0.560
Positive	147 (40.83)	83 (42.35)	64 (39.02)		86 (45.26)	41 (43.16)	45 (47.37)	
Follow-up (months)	59.04 ± 14.33	58.11 ± 14.85	60.16 ± 13.65	0.974	58.83 ± 15.67	57.12 ± 16.37	60.55 ± 14.82	0.496

^a^ Median [IQR]; n (%); $\bar{x}$ ± s.

^b^ Wilcoxon rank sum test; Pearson’s Chi-squared test.

^c^ Abbreviations: BMI, body mass index; NAC, neo-adjuvant chemotherapy; IDC, invasive ductal carcinoma; ER, estrogen receptor; PR, progesterone receptor; HER-2, human epidermal growth factor receptor-2; ALN, axillary lymph node.

### Correlation analysis of RSR intervention with lung metastasis-free survival and disease-free survival in breast cancer

**FIGURE 3:**
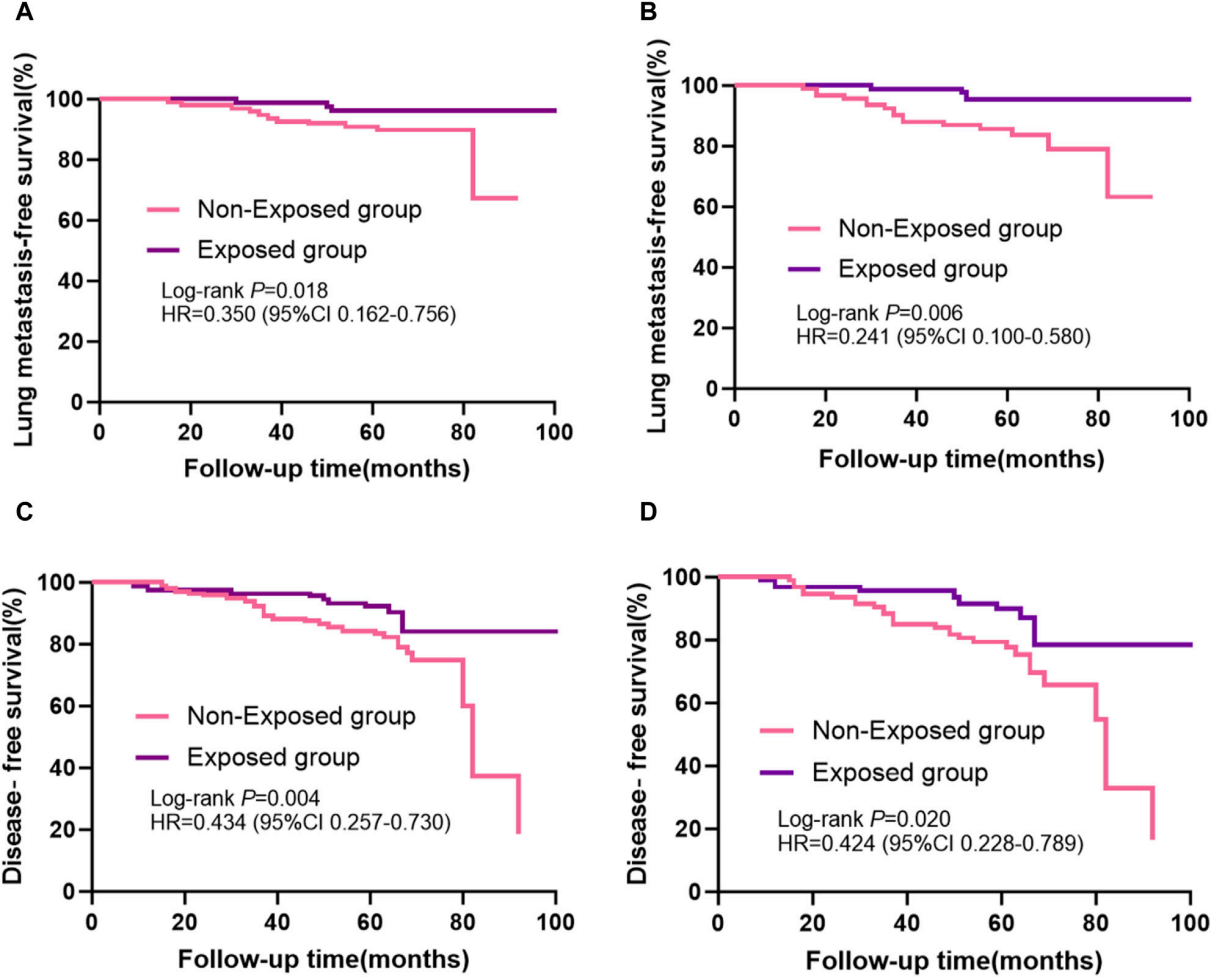
Kaplan-Meier survival curves from patients with breast cancer are shown, including: **(A)** lung metastases-free survival before propensity score matching and **(B)** after propensity score matching; **(C)** disease-free survival before propensity score matching and **(D)** after propensity score matching.

Kaplan-Meier curves for lung metastases of breast cancer before and after PSM are shown in Figures 3A,B, respectively. Disease-free survival (DFS) curves for the breast cancer patients before and after PSM are shown in Figures 3C,D. Patients who received RSR treatment had a higher incidence of lung metastases-free as well as disease-free survival at 5-year postoperative follow-up. As shown in Figures 3B,D, lung metastases and distant metastases occurred later in the exposed group than in the non-exposed group when cumulative lung metastasis-free survival and disease-free survival were the same.

### Cox survival analysis of distant metastases from breast cancer

Cox univariate analysis showed that endocrine therapy, neoadjuvant chemotherapy, ER status, PR status, lymph node metastasis, and RSR therapy were influential factors for the development of distant metastasis in postoperative breast cancer patients (Table 4). Further multivariate analysis showed that neoadjuvant chemotherapy and lymph node metastasis were independent risk factors for the development of breast cancer distant metastasis, with risk ratios of 11.783 (95% *CI*: 4.835–28.717) and 3.175 (95% *CI*: 1.558–6.467), respectively, while endocrine and RSR treatments were independent protective factors against the development of breast cancer distant metastasis. The risk ratios for endocrine and RSR treatments were 0.046 (95% *CI*: 0.005–0.418) and 0.473 (95% *CI*: 0.233–0.959), respectively (Table 5).

**TABLE 4:** Results of Cox univariate analysis for distant metastases from breast cancer.

Variable	Distant metastases of breast cancer					
	*B*	*SE*	*Wald*	*p-value*	Exp (*B*)	*95%CI*
Age	0.575	0.333	2.982	0.084	1.777	0.925–3.414
BMI	−0.415	0.419	0.981	0.322	0.660	0.290–1.502
Chemotherapy	3.026	6.524	0.215	0.643	20.615	0.000–7362745.281
Targeted therapy	−0.014	0.418	0.001	0.974	0.987	0.435–2.239
Endocrine therapy	−0.996	0.321	9.616	0.002	0.369	0.196–0.693
Radiotherapy	0.007	0.317	0.001	0.981	1.008	0.541–1.876
NAC	2.000	0.391	26.114	0.000	7.386	3.430–15.903
Breast conservation	0.166	0.339	0.240	0.624	1.181	0.607–2.296
IDC	1.054	0.734	2.062	0.151	2.868	0.681–12.081
Histopathological grade	0.568	0.327	3.013	0.083	1.765	0.929–3.353
ER	−0.906	0.322	7.931	0.005	0.404	0.215–0.759
PR	−1.169	0.444	6.947	0.008	0.311	0.130–0.741
HER2	0.230	0.368	0.391	0.531	1.259	0.612–2.590
Ki67	0.670	0.482	1.927	0.165	1.954	0.759–5.028
ALN	1.029	0.347	8.808	0.003	2.799	1.418–5.524
Exposed	−0.865	0.346	6.236	0.013	0.421	0.214–0.830

Abbreviations: BMI, body mass index; NAC, neo-adjuvant chemotherapy; IDC, invasive ductal carcinoma; ER, estrogen receptor; PR, progesterone receptor; HER-2, human epidermal growth factor receptor-2; ALN, axillary lymph node.

Note: *B* indicates regression coefficients; Exp(*B*) indicates hazard ratio; *SE*, indicates standard errors of regression coefficients; Exposed indicates RSR, treatment; *95%CI*, indicates 95% confidence interval for the hazard ratio.

**TABLE 5:** Results of Cox multivariate analysis for distant metastases from breast cancer.

Variable	Distant metastases of breast cancer					
	*B*	*SE*	*Wald*	*p-value*	Exp (*B*)	*95%CI*
Endocrine therapy	−3.085	1.129	7.471	0.006	0.046	0.005–0.418
NAC	2.467	0.455	29.453	0.000	11.783	4.835–28.717
ER	1.944	1.105	3.094	0.079	6.985	0.801–60.922
PR	−0.585	0.509	1.319	0.251	0.557	0.205–1.511
ALN	1.155	0.363	10.126	0.001	3.175	1.558–6.467
Exposed	−0.750	0.361	4.308	0.038	0.473	0.233–0.959

Abbreviations: NAC, neo-adjuvant chemotherapy; ER, estrogen receptor; PR, progesterone receptor; ALN, axillary lymph node.

Note: *B* indicates regression coefficients; Exp(*B*) indicates hazard ratio; *SE*, indicates standard errors of regression coefficients; Exposed indicates RSR, treatment; *95%CI*, indicates 95% confidence interval for the hazard ratio.

### Cox univariate survival analysis of lung metastases from breast cancer

Cox univariate analysis showed that endocrine therapy, neoadjuvant chemotherapy, ER status, PR status, lymph node metastasis, and RSR therapy were influential factors for the development of lung metastasis in postoperative breast cancer patients (Table 6). Further multivariate analysis showed that neoadjuvant chemotherapy and lymph node metastasis were independent risk factors for the development of breast cancer lung metastasis, with risk ratios of 17.188 (95% *CI*: 5.098–57.944) and 5.812 (95% *CI*: 1.928–17.519), while RSR treatment was an independent protective factor against the development of breast cancer lung metastasis, with a risk ratio of 0.290 (95% *CI*: 0.093–0.908) (Table 7).

**TABLE 6:** Results of Cox univariate analysis for lung metastases from breast cancer.

Variable	Lung metastases of breast cancer					
	*B*	*SE*	*Wald*	*p-value*	Exp (*B*)	*95%CI*
Age	0.980	0.517	3.599	0.058	2.664	0.968–7.333
BMI	−1.092	0.747	2.138	0.144	0.336	0.078–1.450
Chemotherapy	3.026	8.372	0.131	0.718	20.615	0.000–275642554.564
Targeted therapy	−0.267	0.627	0.182	0.670	0.765	0.224–2.616
Endocrine therapy	−1.124	0.451	6.229	0.012	0.325	0.134–0.786
Radiotherapy	−0.121	0.447	0.073	0.787	0.886	0.369–2.130
NAC	2.290	0.499	21.029	0.000	9.875	3.711–26.279
Breast conservation	0.026	0.470	0.003	0.955	1.027	0.409–2.580
IDC	−3.036	6.989	0.189	0.664	0.048	0.000–42655.515
Histopathological grade	0.829	0.472	3.084	0.079	2.290	0.908–5.775
ER	−0.957	0.450	4.519	0.034	0.384	0.159–0.928
PR	−2.381	1.026	5.383	0.020	0.092	0.012–0.691
HER2	−0.483	0.627	0.594	0.441	0.617	0.180–2.108
KI67	0.954	0.748	1.628	0.202	2.597	0.599–11.250
ALN	1.336	0.518	6.661	0.010	3.804	1.379–10.492
Exposed	−1.428	0.560	6.506	0.011	0.240	0.080–0.718

Abbreviations: BMI, body mass index; NAC, neo-adjuvant chemotherapy; IDC, invasive ductal carcinoma; ER, estrogen receptor; PR, progesterone receptor; HER-2, human epidermal growth factor receptor-2; ALN, axillary lymph node.

Note: *B* indicates regression coefficients; Exp(*B*) indicates hazard ratio; *SE*, indicates standard errors of regression coefficients; Exposed indicates RSR, treatment; *95%CI*, indicates 95% confidence interval for the hazard ratio.

**TABLE 7:** Results of Cox multivariate analysis for lung metastases from breast cancer.

Variable	Lung metastases of breast cancer					
	*B*	*SE*	*Wald*	*p-value*	Exp (*B*)	*95%CI*
Endocrine therapy	−11.891	93.952	0.016	0.899	0.000	0.000–6.4178E + 74
NAC	2.844	0.620	21.041	0.000	17.188	5.098–57.944
ER	10.583	93.951	0.013	0.910	39,444.156	0.000–3.6887E + 84
PR	−1.824	1.077	2.870	0.090	0.161	0.020–1.331
ALN	1.760	0.563	9.775	0.002	5.812	1.928–17.519
Exposed	−1.236	0.582	4.519	0.034	0.290	0.093–0.908

Abbreviations: NAC, neo-adjuvant chemotherapy; ER, estrogen receptor; PR, progesterone receptor; ALN, axillary lymph node.

Note: *B* indicates regression coefficients; Exp(*B*) indicates hazard ratio; *SE*, indicates standard errors of regression coefficients; Exposed indicates RSR, treatment; *95%CI*, indicates 95% confidence interval for the hazard ratio.

**FIGURE 4:**
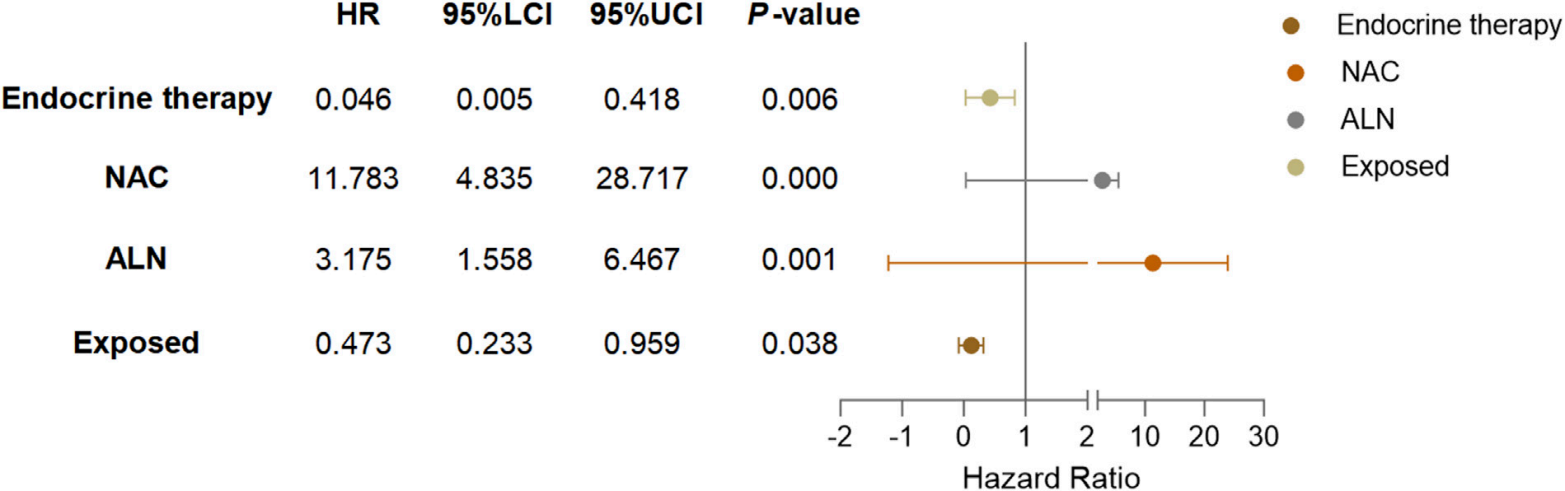
Forest plots illustrate the exploratory analysis of disease-free survival after PSM.

**FIGURE 5:**
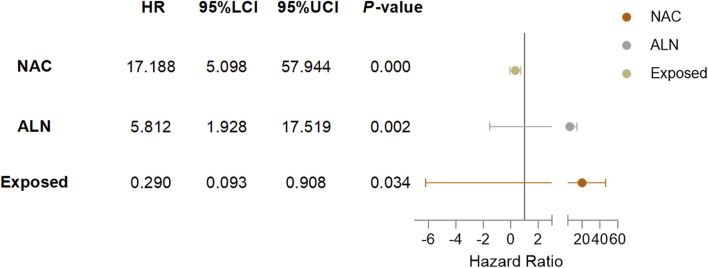
Forest plots illustrate the exploratory analysis of lung metastases-free survival after PSM.

Based on the results of Cox multivariate analysis, we plotted random forest plots for lung metastasis and distant metastasis in breast cancer. Compared with breast cancer patients who did not receive neo-adjuvant chemotherapy or who had lymph node metastases, neo-adjuvant chemotherapy or lymph node metastases significantly increased the risk of distant metastases (11.783-fold and 3.175-fold, respectively) and lung metastases (17.188-fold and 5.812-fold, respectively) (Figures 4, 5). On the other hand, endocrine therapy and RSR intervention significantly reduced the risk of distant metastasis of breast cancer, which were 0.046-fold and 0.473-fold that of patients who did not receive endocrine therapy or RSR intervention, respectively (Figure 4). In addition, our study showed that RSR significantly reduced the risk of developing lung metastases from breast cancer, with the risk of developing lung metastases in the exposed group being only 0.290-fold that of the non-exposed group compared to the non-exposed group (Figure 5).

## Discussion

The results of this study suggest that combined RSR has significant advantages over standard biomedical treatment alone in reducing the incidence of lung and distant metastases in breast cancer patients. The Kaplan-Meier survival analysis curves showed that the exposed group had a higher lung metastasis-free survival rate and a higher disease-free survival rate than the non-exposed group at the same follow-up time points. From the results of the Cox multivariate analysis, it can be seen that the efficacy of RSR in preventing lung metastases from breast cancer is better than that in preventing distant metastases, and the incidence of distant metastases and lung metastases in the exposed group is 0.473-fold and 0.290-fold, respectively, than that of the unexposed group. This means that combined RSR reduces the incidence of breast cancer lung metastases by an additional 26.3% compared to reducing the incidence of distant metastases. This shows that RSR has unique advantages in fighting breast cancer lung metastases.

Current therapeutic strategies for breast cancer lung metastasis focus on inhibiting the formation of pre-metastatic niche (PMN) by targeting the molecular and cellular components of the PMN, thereby preventing tumour cell metastasis (Ding et al., 2022). Myeloid-derived suppressor cells (MDSCs) are a heterogeneous population of myeloid cells, deriving from myeloid progenitor cells and immature myeloid cells (Veglia et al., 2021). MDSCs have been detected in the lungs of mice bearing mammary adenocarcinoma prior to metastatic spread (Yan et al., 2010). MDSCs in the premetastatic niches may facilitate the escape of tumour cells by suppressing immune cells, inducing matrix remodelling and promoting angiogenesis, which in turn facilitate the engraftment of tumour cells (Wang et al., 2019; Veglia et al., 2021). Growing experimental evidence suggests that MDSCs are key determinants of lung PMN formation in mice with breast cancer. Our recent experiments showed that RSR may play a role in inhibiting breast cancer lung metastasis by reducing the recruitment of MDSCs within the lung PMN of 4T1 breast cancer mice (unpublished content). Further transcriptomic results suggested that its mechanism of reducing MDSCs recruitment in lung PMN may be related to the inhibition of the production of various factors such as granulocyte colony-stimulating factor (G-CSF), interleukin-6 (IL-6), interleukin-1β (IL-1β), chemokine (C-X-C Motif) ligand 1 (CXCL1), chemokine (C-X-C Motif) cigand 2 (CXCL2), S100 calcium binding protein A8 (S100A8). (unpublished content). G-CSF is one of the key growth factors that regulate the generation of MDSCs (Pan et al., 2008). It can stimulate the bone marrow to produce immature myeloid cells (IMC), the precursors of MDSCs. Under cancer conditions, large numbers of IMCs can be pathologically activated by tumour-derived factors such as IL-6 and IL-1β to differentiate into MDSCs (Al et al., 2019). Finally, MDSCs are prematurely recruited into the lung PMN to promote the development of lung metastases in response to multiple chemokines, including CXCL1, CXCL2 and S100A8 (Steele et al., 2016). This suggests that inhibiting the generation, activation and recruitment of MDSCs in lung PMN may be one of the key mechanisms by which RSR inhibits breast cancer lung metastasis.

In addition, our previous study showed that RSR reduced matrix metalloproteinase 9 (MMP-9) expression in lung metastases (Ding et al., 2022). Matrix metalloproteinases (MMPs) are a family of zinc-dependent endopeptidases (Verma and Hansch, 2007). There are currently 24 known human MMPs with different structural domains, including collagenases, gelatinases, stromelysins, matrilysins and membrane-type MMPs. MMP-9, MMPs with gelatinase activity, are highly expressed in invasive breast tumours, promote tumour cell migration and are associated with poor prognosis (Pellikainen et al., 2004; Che et al., 2015). In breast cancer lung metastasis, MMP-9 disrupts VE-calmodulin junctions in the extracellular matrix of the pulmonary vascular endothelium, promoting tumour cell extravasation and metastatic nodule formation (Kaplan et al., 2005; Yan et al., 2010). We therefore hypothesised that reducing extracellular matrix remodelling in the pulmonary vascular endothelium by reducing MMP-9 production may also be one of the mechanisms of action of RSR in inhibiting breast cancer lung metastasis.

In conclusion, our study is the first to evaluate the clinical efficacy of RSR for breast cancer lung metastases in the real world by adjusting for confounders using PSM, creating comparable groups of exposed and non-exposed patients, and effectively controlling for baseline confounders to obtain relatively accurate and reliable results. However, this study has several shortcomings: ① Telephone and outpatient follow-up, and the information provided by patient recall are biased from reality; ② The follow-up period was long and there was a difference in the rate of lost visits between the exposed and non-exposed groups, and there may be a lost visit bias; ③ Cox regression is unable to resolve the collinearity between the two variables and may affect the accuracy of the results; ④ This study mainly evaluated the effect of RSR on the occurrence of lung metastases in breast cancer and did not further investigate other common metastatic sites such as bones, organs and brain, which will need to be categorised and described in the future when the sample size is increased.

## Conclusion

In conclusion, the results of this study show that the combined use of RSR in addition to standard biomedical treatment therapy can significantly increase 5-year lung metastasis-free survival and disease-free survival in breast cancer patients and improve the long-term prognosis of patients. Despite some shortcomings, this study provides evidence of the effectiveness of RSR interventions for the specific population of breast cancer lung metastases and has implications for risk factor management in patients. Our team will continue to follow these patients, increasing the sample size and improving the quality of the study to provide more reliable evidence.

## Data Availability

The original contributions presented in the study are included in the article/Supplementary Material, further inquiries can be directed to the corresponding authors.

## References

[B1] Al S. M. F. Amrein M. A. Bührer E. D. Huguenin A. L. Radpour R. Riether C. (2019). T-cell-Secreted TNFα induces emergency myelopoiesis and myeloid-derived suppressor cell differentiation in cancer. Cancer Res. 79 (2), 346–359. 10.1158/0008-5472.CAN-17-3026 30389698

[B2] Berman A. T. Thukral A. D. Hwang W. T. Solin L. J. Vapiwala N. (2013). Incidence and patterns of distant metastases for patients with early-stage breast cancer after breast conservation treatment. Clin. Breast Cancer 13 (2), 88–94. 10.1016/j.clbc.2012.11.001 23218473

[B3] Borin T. F. Angara K. Rashid M. Shankar A. Iskander A. Ara R. (2017). Abstract 1043: CSF-1R inhibitor prevented pre-metastatic lung niches in metastatic mammary tumor. Cancer Res. 77, 1043. 10.1158/1538-7445.AM2017-1043

[B4] Che Y. L. Luo S. J. Li G. Cheng M. Gao Y. M. Li X. M. (2015). The c3g/rap1 pathway promotes secretion of mmp-2 and mmp-9 and is involved in serous ovarian cancer metastasis. Cancer Lett. 359 (2), 241–249. 10.1016/j.canlet.2015.01.019 25617801

[B5] Ding S. Q. Wu X. Q. Shao S. J. (2022). Effects of Ru’ai Shuhou Formula on lung metastasis of breast cancer in 4T1 mice through SDF-1/CXCR4 signaling pathway China Journal of Traditional Chinese Medicine and Pharmacy. Chin. J. Tradit. Chin. Med. Pharm. 37 (06), 3574–3577.

[B6] EL Baiomy M. A. El Kashef W. F. (2017). ERCC1 expression in metastatic triple negative breast cancer patients treated with platinum-based chemotherapy. Asian Pac J. Cancer Prev. 18 (2), 507–513. 10.22034/APJCP.2017.18.2.507 28345838 PMC5454751

[B7] Gao Q. Q. Wan H. Li X. R. (2022). Effects of“Recipe after Breast Cancer Operation”on disease free survival of breast cancer patients after mastectomy. Shanghai J. Traditional Chin. Med. 45 (10), 49–52.

[B8] Giaquinto A. N. Sung H. Miller K. D. Kramer J. L. Newman L. A. Minihan A. (2022). Breast cancer statistics, 2022. CA Cancer J. Clin. 72 (6), 524–541. 10.3322/caac.21754 36190501

[B9] Kaplan R. N. Riba R. D. Zacharoulis S. Bramley A. H. Vincent L. Costa C. (2005). Vegfr1-positive haematopoietic bone marrow progenitors initiate the pre-metastatic niche. Nature 438 (7069), 820–827. 10.1038/nature04186 16341007 PMC2945882

[B10] Kazmi S. Chatterjee D. Raju D. Hauser R. Kaufman P. A. (2020). Overall survival analysis in patients with metastatic breast cancer and liver or lung metastases treated with eribulin, gemcitabine, or capecitabine. Breast Cancer Res. Treat. 184 (2), 559–565. 10.1007/s10549-020-05867-0 32808239 PMC7599186

[B11] Kroeze S. G. C. Pavic M. Stellamans K. Lievens Y. Becherini C. Scorsetti M. (2023). Metastases-directed stereotactic body radiotherapy in combination with targeted therapy or immunotherapy: systematic review and consensus recommendations by the EORTC-ESTRO OligoCare consortium. Lancet Oncol. 24 (3), e121–e132. 10.1016/S1470-2045(22)00752-5 36858728

[B12] Lee S. H. Diamond M. A. Chadderton A. Liu H. Volgina A. Roman V. (2018). Abstract 3929: the FAD-directed LSD1 specific inhibitor, INCB059872, inhibits cell migration and metastasis by suppressing premetastatic niche formation in a spontaneous metastasis mouse model. Cancer Res. 78, 3929. 10.1158/1538-7445.am2018-3929

[B13] Li J. Wang D. D. Zhao Y. N. Zhou J. W. Tang J. H. (2019a). Clinical assessment of magnetic resonance imaging-guided radiofrequency ablation for breast cancer. Mol. Clin. Oncol. 11 (4), 411–415. 10.3892/mco.2019.1905 31475070 PMC6713947

[B14] Li Z. Wang S. Yang L. Yuan X. H. Suo F. Z. Yu B. (2019b). Experience-based discovery (ebd) of aryl hydrazines as new scaffolds for the development of lsd1/kdm1a inhibitors. Eur. J. Med. Chem. 1 (166), 432–444. 10.1016/j.ejmech.2019.01.075 30739825

[B15] MacDonald I. C. Groom A. C. Chambers A. F. (2002). Cancer spread and micrometastasis development: quantitative approaches for *in vivo* models. Bioessays 24 (10), 885–893. 10.1002/bies.10156 12325121

[B16] Macherey S. Mallmann P. Malter W. Doerr F. Heldwein M. Wahlers T. (2017). Lung metastasectomy for pulmonary metastatic breast carcinoma. Geburtshilfe Frauenheilkd 77 (6), 645–650. 10.1055/s-0043-108252 28769127 PMC5489404

[B17] Medeiros B. Allan A. L. (2019). Molecular mechanisms of breast cancer metastasis to the lung: clinical and experimental perspectives. Int. J. Mol. Sci. 20 (9), 2272. 10.3390/ijms20092272 31071959 PMC6540248

[B18] Pan P. Y. Wang G. X. Yin B. Ozao J. Ku T. Divino C. M. (2008). Reversion of immune tolerance in advanced malignancy: modulation of myeloid-derived suppressor cell development by blockade of stem-cell factor function. Blood 111 (1), 219–228. 10.1182/blood-2007-04-086835 17885078 PMC2200807

[B19] Pellikainen J. M. Ropponen K. M. Kataja V. V. Kellokoski J. K. Eskelinen M. J. Kosma V. M. (2004). Expression of matrix metalloproteinase (mmp)-2 and mmp-9 in breast cancer with a special reference to activator protein-2, her2, and prognosis. Clin. Cancer Res. 10 (22), 7621–7628. 10.1158/1078-0432.CCR-04-1061 15569994

[B20] Qu W. C. Wu X. Q. Feng J. M. (2012). Effect of compound traditional Chinese herbal medicine on rate of five year disease free survival and overall survival in breast cancer patients. China Med. Her. 9 (36), 120–122.

[B21] Siegel R. L. Miller K. D. Wagle N. S. Jemal A. (2023). Cancer statistics, 2023. CA Cancer J. Clin. 73 (1), 17–48. 10.3322/caac.21763 36633525

[B22] Steele C. W. Karim S. A. Leach J. D. G. Bailey P. Upstill-Goddard R. Rishi L. (2016). CXCR2 inhibition profoundly suppresses metastases and augments immunotherapy in pancreatic ductal adenocarcinoma. Cancer Cell 29 (6), 832–845. 10.1016/j.ccell.2016.04.014 27265504 PMC4912354

[B23] Stott S. L. Hsu C. H. Tsukrov D. I. Yu M. Miyamoto D. T. Waltman B. A. (2010). Isolation of circulating tumor cells using a microvortex-generating herringbone-chip. Proc. Natl. Acad. Sci. U. S. A. 107 (43), 18392–18397. 10.1073/pnas.1012539107 20930119 PMC2972993

[B24] The Society of Breast Cancer China Anti-Cancer Association (2015). Guidelines for breast cancer diagnosis and treatment by China Anti-cancer Association (2015 edition). China Onoology 25 (09), 692–754.

[B25] Veglia F. Sanseviero E. Gabrilovich D. I. (2021). Myeloid-derived suppressor cells in the era of increasing myeloid cell diversity. Nat. Rev. Immunol. 21 (8), 485–498. 10.1038/s41577-020-00490-y 33526920 PMC7849958

[B26] Verma R. P. Hansch C. (2007). Matrix metalloproteinases (mmps): chemical-biological functions and (q)SARS. Bioorg Med. Chem. 15 (6), 2223–2268. 10.1016/j.bmc.2007.01.011 17275314

[B27] Waks A. G. Winer E. P. (2019). Breast cancer treatment: a review. JAMA 321 (3), 288–300. 10.1001/jama.2018.19323 30667505

[B28] Wang Y. Ding Y. Guo N. Wang S. (2019). Mdscs: key criminals of tumor pre-metastatic niche formation. Front. Immunol. 10, 172. 10.3389/fimmu.2019.00172 30792719 PMC6374299

[B29] Wu Q. Li J. Zhu S. Wu J. Chen C. Liu Q. (2017). Breast cancer subtypes predict the preferential site of distant metastases: a SEER based study. Oncotarget 8 (17), 27990–27996. 10.18632/oncotarget.15856 28427196 PMC5438624

[B30] Wu X. Q. Que H. F. He C. M. (2000). 37 cases of distant metastatic breast cancer treated by Prof Lu Deming. Acta Univ. Tradit. Medicalis Sin. Pharmacol. Shanghai. 2000 (01), 24–26.

[B31] Wu X. Q. Wan H. Li X. R. (2010). Effect of Ru'ai Shuhou Recipe on immune response in HER2/neu transgenic mice undergoing breast cancer carcinogenesis process. Chin. J. Integr. Traditional Stand. Biomed. Treat. 30 (07), 717–719+756. 20929129

[B32] Xiao W. Zheng S. Liu P. Zou Y. Xie X. Yu P. (2018). Risk factors and survival outcomes in patients with breast cancer and lung metastasis: a population-based study. Cancer Med. 7 (3), 922–930. 10.1002/cam4.1370 29473333 PMC5852337

[B33] Yan H. H. Pickup M. Pang Y. Gorska A. E. Li Z. Chytil A. (2010). Gr-1+CD11b+ myeloid cells tip the balance of immune protection to tumor promotion in the premetastatic lung. Cancer Res. 70 (15), 6139–6149. 10.1158/0008-5472.CAN-10-0706 20631080 PMC4675145

